# Clinical Application Value of Pharmacokinetic Parameters of Vancomycin in Children Treated in the Pediatric Intensive Care Unit

**DOI:** 10.3389/fped.2022.867712

**Published:** 2022-06-30

**Authors:** Bo Zhou, Wenyi Xiong, Ke Bai, Hongxing Dang, Jing Li, Feng Xu, Yue-qiang Fu, Chengjun Liu

**Affiliations:** ^1^Department of Pharmacy, Children’s Hospital of Chongqing Medical University, Chongqing, China; ^2^National Clinical Research Center for Child Health and Disorders, Chongqing, China; ^3^Ministry of Education Key Laboratory of Child Development and Disorders, Chongqing, China; ^4^Chongqing Key Laboratory of Pediatrics, Chongqing, China; ^5^Department of Pediatrics, Chengdu Seventh People’s Hospital, Chengdu Tumor Hospital, Chengdu, China; ^6^Department of Critical Care Medicine, Children’s Hospital of Chongqing Medical University, Chongqing, China

**Keywords:** nephrotoxicity, children, therapeutic drug monitoring, pharmacokinetics/pharmacodynamics, vancomycin

## Abstract

**Objective:**

To explore the efficacy and safety of vancomycin as measured by pharmacokinetic/pharmacodynamic parameters in children with severe infection in the Pediatric Intensive Care Unit (PICU) and to determine the appropriate threshold for avoiding nephrotoxicity.

**Methods:**

The medical records of hospitalized children with severe infection treated with vancomycin in the PICU of a tertiary pediatric hospital from September 2018 to January 2021 were retrospectively collected. Univariate analysis was used to assess the correlation between vancomycin pharmacokinetic/pharmacodynamic parameters and therapeutic efficacy or vancomycin-related nephrotoxicity. Binary logistic regression was used to analyze the risk factors for vancomycin-related nephrotoxicity. The vancomycin area under the concentration-time curve over 24 h (AUC_0–24_) threshold was determined by receiver operating characteristic (ROC) curve analysis.

**Results:**

One hundred and 10 patients were included in this study. Seventy-six patients (69.1%) exhibited clinically effective response, while the rest exhibited clinically ineffective response. There were no significant differences in APACHE II score, steady-state trough concentration, peak concentration or AUC_0–24_ of vancomycin between the effective and ineffective groups. Among the 110 patients, vancomycin-related nephrotoxicity occurred in 15 patients (13.6%). Multivariate analysis showed that vancomycin treatment duration, trough concentration, and AUC_0–24_ were risk factors for vancomycin-related nephrotoxicity. The ROC curve indicated that AUC_0–24_ < 537.18 mg.h/L was a suitable cutoff point for predicting vancomycin-related nephrotoxicity.

**Conclusion:**

No significant correlations were found between the trough concentration or AUC_0–24_ of vancomycin and therapeutic efficacy when the daily dose of vancomycin was approximately 40 mg/kg d, while the trough concentration and AUC_0–24_ were both closely related to vancomycin-related nephrotoxicity. The combination of AUC_0–24_ and trough concentration for therapeutic drug monitoring may reduce the risk of nephrotoxicity.

## Introduction

Gram-positive bacteria (GPB) have become the primary cause of severe infection in children, especially the emergence of multiple drug-resistant bacteria, such as methicillin-resistant Staphylococcus aureus (MRSA), which brings great challenges to the choice of antibacterial drugs. Vancomycin is the primary drug of choice for the treatment of severe Gram-positive bacterial infections, such as MRSA; however, improper application will lead to poor clinical treatment effects and may lead to adverse drug reactions, including rash (1), ototoxicity (2, 3), and nephrotoxicity (4–7). At present, studies and guidelines on the therapy, therapeutic drug monitoring (TDM) and side effects of vancomycin mostly focus on adults, while there are relatively few studies in pediatric populations.

Children are constantly growing, and visceral function is not yet mature, which leads to the metabolism of the drug in the body not being the same as that of adults and making them more likely to induce adverse drug reactions. The relationship between PK/PD parameters of vancomycin and clinical efficacy is not entirely clear in pediatric populations, and some viewpoints of studies regarding vancomycin are conflicting. Some studies have shown that a higher initial trough concentration achieves better outcomes with vancomycin treatment in pediatric patients (8, 9). However, Yoo et al. (10) indicated that the initial trough concentration was not associated with 30-day.

In addition to clinical efficacy, the relationship between vancomycin and renal injury is another focus of pediatricians. Higher serum trough levels of vancomycin have been associated with AKI in pediatric patients (7, 11, 12). However, Moffett et al. (13) found that serum vancomycin concentrations did not predict vancomycin-associated AKI in the pediatric population. Although vancomycin has been used in severe pediatric infections for a long time, an analysis of vancomycin TDM is warranted.

TDM based on PK/PD parameters may improve clinical efficacy and reduce adverse reactions, which is of great significance for the rational use of vancomycin in children with severe infection (14). In the present study, we analyzed the relationship between PK/PD (the trough concentration and the area under the 24-h drug concentration-time curve (AUC_0–24_) of vancomycin) and efficacy and nephrotoxicity. We hypothesized that PK/PD parameters of vancomycin could predict nephrotoxicity.

## Materials and Methods

### Patient Population

Children with severe infection who received intravenous vancomycin treatment and underwent serum concentration (trough concentration and peak concentration) monitoring in the Pediatric Intensive Care Unit (PICU) at Children’s Hospital of Chongqing Medical University from September 2018 to January 2021 were retrospectively identified and included in this study. The Institutional Review Board of Children’s Hospital, Chongqing Medical University approved the study with a waiver of informed consent given that the data were analyzed anonymously, because there were no interventions performed as part of this retrospective study.

The inclusion criteria were as follows: (1) patients aged from 1 month to 18 years old; (2) a usage time of vancomycin ≥ 72 h with serum vancomycin (trough concentration and peak concentration) measurements available; and (3) renal function was normal before vancomycin treatment (normal range of serum creatinine in the Clinical Laboratory of Children’s Hospital of Chongqing Medical University: 15.4–90.4 μmol/L using dry chemistry method; 14–60 μmol/L using enzymatic method).

The exclusion criteria were as follows: (1) patients were treated with other antibacterial drugs within 72 h before the use of vancomycin, such as teicoplanin, linezolid and rifampin; (2) vancomycin was not intravenously administered; (3) blood purification was conducted during vancomycin treatment; (4) there was concomitant use of other nephrotoxic drugs, such as amphotericin B, methotrexate, cyclophosphamide, cyclosporine and tacrolimus; and/or (5) clinical data were missing multiple variables.

### Data Collection

Basic information including age, sex, height, weight, infection site, and primary diagnosis were collected, as well as clinical manifestations, symptoms and signs, blood laboratory tests and imaging examinations before and after vancomycin administration. Meanwhile, data on vancomycin administration (including initial dose, adjustment of administration plan, intravenous infusion time, treatment drug monitoring results), treatment course, duration of mechanical ventilation, length of stay (LOS) in the PICU, LOS in the hospital and mortality.

### Methods of Vancomycin Trough/Peak Concentration Determination

All children included in this study were administered intravenous vancomycin (trade name: Vancocin, 500 mg/bottle, Lilly Suzhou Pharmaceutical Co., Ltd.) for at least 60 min at each administration. According to the Experts’ Consensus on Monitoring Therapeutic Drugs for Children (14), within 30 min to 1 h after the fourth vancomycin infusion, 2 ml venous blood samples were collected and labeled as peak concentration blood samples, and another of 2 ml venous blood sample was collected 30 min before the fifth vancomycin infusion and marked as a valley concentration blood sample. Patients who underwent adjustments to the dosing regimen were required to monitor the serum drug concentration again after the fourth treatment with the adjusted dose. The serum concentration of vancomycin was assessed using chemiluminescent enzyme immunoassay.

### Calculation Method of AUC_0–24_

**FIGURE 1:**
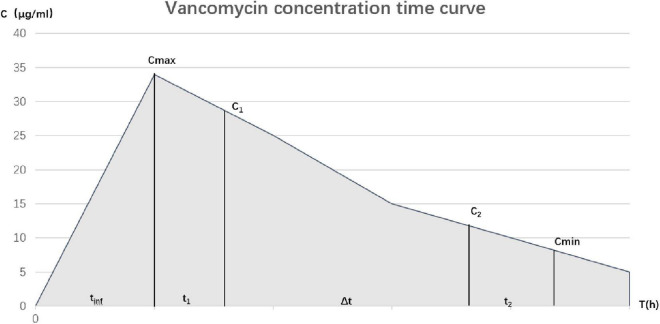
Time curve of vancomycin concentration.


A method based on the primary rate cancelation equation was used to calculate AUC_0–24_ (Figure 1, Equation 1) (15):

(1) $$K_{e}=\frac{Ln\left(C_{1}/C_{2}\right)\;}{\Delta t}{\text{C}}_{\text{mac}}=\frac{{\text{C}}_{1}}{{\text{e}}^{-\mathrm{Ke}\cdot \text{tl}}}\;{\text{C}}_{\min}={\text{C}}_{2}\cdot {\text{e}}^{-\mathrm{Ke}\times \mathrm{t2}}$$

$$AUC_{0-24}=\left(\frac{t_{\inf}\times \left(C_{\max}+C_{\min}\right)}{2}+\frac{\left(C_{\max}-C_{\min}\right)}{K_{\text{e}}}\right)$$

×Numberofdose/day

C_1_ is the measured peak concentration, C_2_ is a measurement of trough concentration, Δt is the time period (in hours) between the two serum concentrations, t_inf_ represents the infusion time, t_1_ is the time between the end of infusion and collection of C_1_, and t_2_ is the time between C_2_ acquisition and the next infusion. The true peak concentration (C_max_) and trough concentration (C_min_) were extrapolated in the reverse and forward directions by K_e_, respectively. AUC_0–24_ was calculated using the modified trapezoidal rule.

### Efficacy Definition

The Acute Physiology and Chronic Health Evaluation II (APACHE II) score was used to quantify the severity of disease (calculated by the worst physiological parameters within 24 h after entering the PICU). The clinical efficacy of treatment in patients was primarily evaluated through the clinical manifestations and laboratory and imaging findings. Clinical efficacy was divided into three levels: cure, improved and ineffective. Patients whose temperature, inflammatory indicators (such as white blood cells, neutrophil percentage, and procalcitonin) and imaging examinations returned to normal and whose bacterial culture turned negative after vancomycin treatment were defined as cured. Compared to before treatment, if the above evaluation indices improved but did not return to normal levels, and the bacterial culture turned negative, this was regarded as improvement. Both cure and improved are considered clinically effective. If after treatment, all evaluation indices showed no significant improvement or even aggravation that needed additional antibacterial drugs, such as linezolid and teicoplanin, or/and the bacterial culture was still positive, this was defined as ineffective.

### Definition of Renal Toxicity

Vancomycin-related nephrotoxicity was defined as at least two consecutive renal function tests suggesting an increase in serum creatinine concentration [absolute increase greater than 0.5 mg/dl (44.2 μmol/l) or more than 50% of the baseline level] in a patient after vancomycin treatment and cannot be explained by other reasons (16).

### Statistical Analysis

SPSS 26.0 software (SPSS Inc., Chicago, IL, United States) was used for statistical processing, and all variables were summarized using descriptive statistics. The mean ± standard deviation ($\bar{x}$ ± s) was adopted for normal distribution of continuous variables, and the median (25% percentile and 75% percentile) was adopted for skewed distribution [*M*(P25,P75)]. Counts and percentages (%) were used to describe categorical variables. For univariate analysis, the *t*-test was used for normal distribution, and Mann-Whitney *U*-test was used for skewed distribution of continuous variables. Comparison of categorical variables was performed by the chi-square test. Multivariate analysis was performed using binary logistic regression. All statistical tests were performed bilaterally, and the difference was considered statistically significant if *P* < 0.05.

## Results

### Clinical Data

A total of 110 patients were retrospectively enrolled, and their clinical characteristics are shown in Table 1. Eighty-four cases (76.4%) among the 110 children were discharged from the hospital, and 76 cases (69.1%) were considered effective after treatment with vancomycin according to the definition provided. With no ototoxicity occurring, the most common adverse reactions among all patients were nephrotoxicity (13.6%) and rash (6.4%). Median steady-state daily dose of vancomycin was 40.00 mg/kg d (IQR: 40.00, 58.71). The steady-state daily dose of 74.5% (82/110) children ranged from 40 to 60 mg/kg.d. Good outcome refers to recovery from the disease and discharge from the hospital, and poor outcome refers to treatment failure and subsequent death in the hospital.

**TABLE 1:** Demographic and clinical characteristics of the patients.

Characteristic	All patients (*n* = 110)
Gender	
Male, *n* (%)	57 (51.8%)
Female, *n* (%)	53 (48.2%)
Age (years)	
< 1, *n* (%)	35 (31.8%)
1–3, *n* (%)	28 (25.5%)
4–7, *n* (%)	23 (20.9%)
8–12, *n* (%)	17 (15.5%)
> 12, *n* (%)	7 (6.4%)
Weight (kg), IQR	12.0 (8.0, 22.1)
Serum creatinine (μmol/L), IQR	28.6 (20.8, 42.9)
Steady-state daily dose (mg/kg d), IQR	40.00 (40.00, 58.71)
Treatment outcome	
Good outcome, *n* (%)	84 (76.4%)
Poor outcome, *n* (%)	26 (23.6%)
Clinical efficacy of vancomycin	
Effective, *n* (%)	76 (69.1%)
Ineffective, *n* (%)	34 (30.9%)
Adverse reactions	
Nephrotoxicity, *n* (%)	15 (13.6%)
Rash, *n* (%)	7 (6.4%)

*IQR, interquartile range.*

### Analysis of the Clinical Efficacy of Vancomycin

According to the efficacy criteria, 110 patients were divided into effective and ineffective groups. There were no significant differences in APACHE II scores, steady-state daily dose, steady-state trough/peak concentrations, AUC_0–24_, length of vancomycin therapy, LOS in the hospital, LOS in the ICU, or mechanical ventilation time between the effective and ineffective groups (Table 2).

**TABLE 2:** Univariate analysis results between the effective and ineffective groups.

Variable	Effective group	Ineffective group	*t/Z-*value	*P-*value
*n* (%)	76 (69.1%)	34 (30.9%)		
APACHE II score (± *s*)	30 ± 7	29 ± 8	0.419	0.676
Steady-state daily dose (mg/kg d), IQR	40.00 (40.00, 57.06)	40.61 (40.00, 60.00)	1.923	0.054
Steady-state trough concentration (mg/L), IQR	9.56 (6.15, 13.70)	10.71 (7.18, 16.51)	1.410	0.159
Steady-state peak concentration (mg/L), IQR	25.16 (20.33, 33.26)	25.43 (20.03, 34.29)	0.158	0.874
AUC_0–24_ (mg.h/L), IQR	503.53 (386.02, 609.17)	480.74 (386.48, 779.11)	0.647	0.518
Length of vancomycin therapy (days), IQR	10 (7, 15)	10 (6, 18)	0.230	0.818
LOS of hospital (days), IQR	29 (20, 47)	27 (16, 38)	1.598	0.110
LOS of ICU (days), IQR	11 (7, 18)	12 (8, 19)	0.081	0.935
Mechanical ventilation time (hours), IQR	169 (90, 314)	172 (84, 478)	0.010	0.992

*Data shown are the mean ± standard deviation or median (interquartile range), ICU, intensive care unit; IQR, interquartile range; LOS, length of stay.*

Gram-positive bacteria were detected in 66 of 110 children 83 times in total. The 66 patients with detected pathogens were divided into two groups according to whether the pathogen was cleared after vancomycin treatment. The drug sensitivity results showed that the MICs of vancomycin for Gram-positive bacteria detection were 0.5 mg/L (51/83, 61.4%), 1 mg/L (26/83, 31.3%), and 2 mg/L (6/83, 7.2%). The APACHE II score, steady-state daily dose, steady-state trough/peak concentration, AUC_0–24_/MIC, and medication course were also compared between the two groups, and the results are shown in Table 3. There were no statistically significant differences in the disease severity, medication, therapeutic drug monitoring, or treatment outcome between the two groups.

**TABLE 3:** Univariate analysis results between the pathogen-cleared and pathogen-uncleared groups.

Variable	Pathogen-cleared group	Pathogen-uncleared group	*t/Z-*value	*P-*value
*n* (%)	49 (74.2%)	17 (25.8%)		
APACHE II score (x̄± s)	29 ± 5	29 ± 5	0.341	0.735
Steady-state daily dose (mg/kg d), IQR	42.11 (40.00, 59.23)	40.00 (40.00, 60.00)	0.023	0.982
Steady-state trough concentration (mg/L), IQR	11.12 (7.12, 13.83)	7.70 (5.75, 10.71)	1.628	0.104
Steady-state peak concentration (mg/L), IQR	26.37 (20.19, 32.34)	24.30 (19.28, 29.87)	0.784	0.433
AUC_0–24_/MIC (mg.h/L), IQR	833.02 (562.65, 1058.96)	622.47 (433.71, 1032.42)	1.063	0.288
Length of vancomycin therapy (days), IQR	10 (7, 17)	13 (5, 22)	0.874	0.382

*Data shown are the mean ± standard deviation or median (interquartile range), IQR, interquartile range.*

### Analysis of Vancomycin Nephrotoxicity

According to the renal toxicity criteria, 110 children were divided into nephrotoxic and non-nephrotoxic groups. The clinical characteristics of the two groups are compared in Table 4. The differences in APACHE II scores, single dose and daily dose at steady state, trough/peak concentrations at steady state and AUC_0–24_ between the two groups were all statistically significant.

**TABLE 4:** Univariate analysis results between the nephrotoxic and non-nephrotoxic groups.

Variable	Non-nephrotoxic group	Nephrotoxic group	*t/Z-*value	*P-*value
No. (%)	95 (86.4%)	15 (13.6%)		
APACHE II score (x̄± s)	29 ± 7	36 ± 7	3.791	0.001
Steady-state single dose (mg/kg)	10.00 (10.00, 14.91)	10.00 (7.14, 10.00)	3.274	0.001
Steady-state daily dose (mg/kg•d)	40.00 (40.00, 59.64)	40.00 (28.57, 40.00)	3.209	0.001
Steady-state trough concentration (mg/L), IQR	9.33 (6.09, 12.94)	16.41 (11.59, 34.24)	4.277	<0.001
Steady-state peak concentration (mg/L), IQR	25.32 (20.06, 30.93)	33.6 (23.23, 45.86)	2.604	0.009
AUC_0–24_/MIC (mg•h/L), IQR	478.80 (374.34, 579.55)	763.24 (464.81, 1297.89)	3.401	0.001
Length of vancomycin therapy (days), IQR	10 (7, 15)	10 (7, 14)	0.161	0.872

*IQR, interquartile range.*

Variables such as age, sex, weight, and the above clinical characteristics were included in the binary logistic regression. Due to the multiple collinearity relationships between the daily dose and the steady-state single dose, AUC_0–24_ and the steady-state trough/peak concentration, the steady-state trough/peak concentration or AUC_0–24_ and age, sex, weight, Apache II score, steady-state daily dose and vancomycin treatment time were selected for the binary logistic regression analysis, which was shown in Table 5. Multivariate logistic analysis revealed that vancomycin treatment time, trough concentration and AUC_0–24_ were independent risk factors for nephrotoxicity.

**TABLE 5:** Multivariate logistic analysis of nephrotoxicity in the AUC_0–24_ and trough concentration models.

Variable	AUC_0–24_ model		Trough concentration model	
	*OR* (95% CI)	*P-*value	*OR* (95% CI)	*P-*value
Daily dose (mg/kg d)	0.851 (0.784–0.925)	<0.001	0.872 (0.805–0.944)	0.001
Length of vancomycin therapy (days)	1.099 (0.925–1.202)	0.040	1.102 (1.007–1.206)	0.035
AUC_0–24_	1.005 (1.003–1.008)	<0.001	–	–
Steady-state trough concentration (mg/L)	–	–	1.176 (1.087–1.272)	<0.001

*CI- confidence interval; OR- odds ratio.*

**FIGURE 2:**
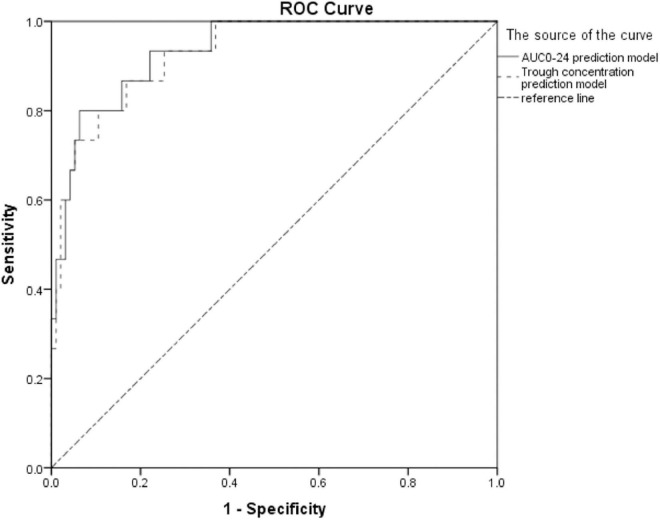
ROC curves of prediction probability and vancomycin nephrotoxicity of two models (the area under the ROC curve of the AUC_0–24_ model was 0.935, 95% CI: 0.878–0.992, *P* < 0.001; the area under the ROC curve of the trough concentration model was 0.928, 95% CI: 0.868–0.989, *P* < 0.001).

Curves of prediction probability and receiver operating characteristic (ROC) of renal toxicity of the two prediction models where AUC_0–24_ and trough concentration were drawn (Figure 2), showing that both models effectively predicted renal toxicity. The area under the ROC curve of the AUC_0–24_ model was larger than that of the trough concentration model, but there was no statistically significant difference between them.

**FIGURE 3:**
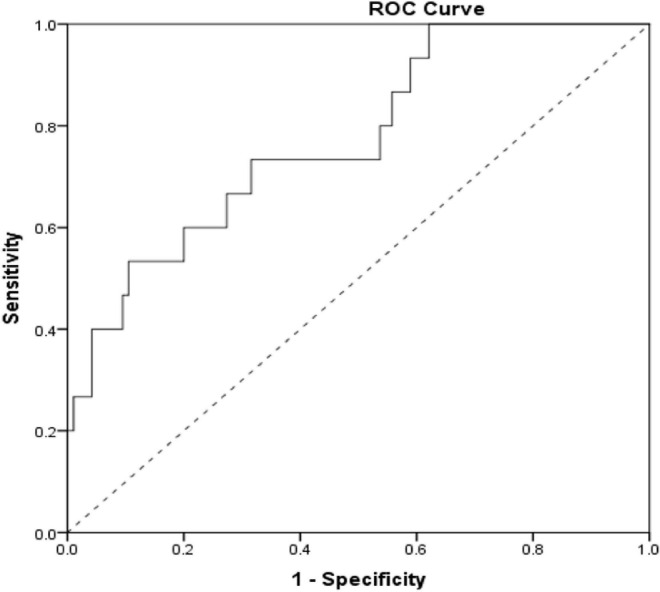
ROC curve of the AUC_0–24_ prediction level of vancomycin-related nephrotoxicity (the area under ROC curve = 0.774, 95% CI: 0.648–0.900, *P* = 0.001).

According to the ROC curve of AUC_0–24_ and nephrotoxicity (Figure 3), vancomycin AUC_0–24_ was demonstrated to be a tool for predicting the risk of vancomycin nephrotoxicity, and 537.18 mg.h/L might be a suitable threshold. The sensitivity and specificity for predicting vancomycin-related nephrotoxicity were 73 and 68%, respectively, and if the AUC_0–24_ exceeded 537.18 mg.h/L, the risk of nephrotoxicity was significantly increased.

### Correlation Between Trough Concentration and AUC_0–24_

In 2015, pediatric experts in China reached a consensus on therapeutic drug monitoring (14) that the suggested monitoring range of vancomycin trough concentration should be 5–10 mg/L for children and 10–20 mg/L for severely infected children. When the trough concentration was above 20 mg/L, nephrotoxicity was more likely to occur (14). Nevertheless, the vancomycin monitoring guidelines of the American Association of Infectious Diseases in 2020 and the evidence-based guidelines for therapeutic drug monitoring of vancomycin (2020 Update by the Division of Therapeutic Drug Monitoring, Chinese Pharmacological Society) recommended keeping AUC_0–24_/MIC in the range of 400–600, which means AUC_0–24_ should be in the range of 400–600 (mg•h/L), assuming that vancomycin MIC was 1 mg/L (17, 18). The ranges of AUC_0–24_ corresponding to three steady-state trough concentration ranges (<10 mg/L, 10–20 mg/L, > 20 mg/L) were analyzed (Table 6). Only 31 of 110 children failed to reach the range of AUC_0–24_ > 400 mg.h/L (Table 6). In addition, it can be seen from the re-analysis of the data in Table 6. That 51.0% (25/49) of children with AUC_0–24_ ranging from 400 to 600 mg.h/L exhibited trough concentrations < 10 mg/L; 37.5% (15/40) of children with trough concentrations ranging from 10 to 20 mg/L had AUC_0–24_ ranging from > 600 mg.h/L.

**TABLE 6:** Proportion of AUC_0–24_ in different trough concentration ranges.

No. (%)	AUC_0–24_ range (mg.h/L)			Total
	<400	400–600	>600	
Trough concentration < 10 mg/L, *n* (%)	30 (27.3%)	25 (22.7%)	3 (2.7%)	58 (52.7%)
10 mg/L ≤ trough concentration ≤ 20 mg/L, *n* (%)	1 (0.9%)	24 (21.8%)	15 (13.6%)	40 (36.4%)
Trough concentration > 20 mg/L, *n* (%)	0 (0)	0 (0)	12 (10.9%)	12 (10.9%)
Total	31 (28.2%)	49 (44.5%)	30 (27.3)	110 (100%)

## Discussion

Vancomycin is widely used in critically ill children suspected of Gram-positive bacterial infection. Due to the characteristics of vancomycin, it is important to monitor its use according to vancomycin PK/PD parameters, and the trough concentration remains the primary indicator that is valued and monitored in the clinic. The consensus of Chinese experts on the clinical application of vancomycin in 2011 (19) and the guidelines for the treatment of MRSA infection conducted by the American Society of Infectious Diseases (20) both recommended maintaining the trough concentration at 15–20 mg/L in children with severe infection. However, it is worth noting that most evidence for these recommendations is derived from adult data.

In recent years, pediatric studies have found that the vancomycin trough concentrations needed to achieve the target AUC/MIC were different than the adult goal troughs (21–25). One qualitative systematic review indicated that trough concentrations of 6–10 mg/l were likely sufficient to achieve AUC/MIC ≥ 400 in pediatric patients (26). Targeting trough concentrations > 15 μg/ml would lead to overshooting the target AUC_0–24 *h*_ above 400 and increased the risk of nephrotoxicity and other adverse events (22, 23). Furthermore, the correlation between the vancomycin dose given and the ideal trough concentration achieved was poor (27). In the nephrotoxic group in our study, trough concentrations were 16.41 (11.59, 34.24) mg/L at a median dose of 40 mg/kg d, while the AUC_0–24_ was as high as 763.24 (464.81, 1297.89). This suggested that the significant increase in trough concentration and AUC would lead to nephrotoxicity even at normal daily doses.

The 2020 guidelines for monitoring the efficacy of vancomycin for the treatment of severe MRSA infection (17) proposed that the AUC_0–24_/MIC of patients with severe MRSA infection should be kept in the range of 400–600 rather than monitoring trough concentration only. For pediatric patients with non-invasive MRSA or other infections, no sufficient evidence has been suggested regarding whether trough concentration or AUC_0–24_ is more suitable for vancomycin monitoring.

In this study, the calculation of AUC_0–24_ was based on the first-order rate elimination equation (15), which was one of the most recommended methods at present and can be applied in follow-up due to its accuracy and operability in clinical practice. The clinical results were analyzed for both curative effect and bacterial clearance, making the determination of clinical efficiency reliable. Multiple previous cohort studies (28–31) have revealed that TDM with AUC_0–24_ in adults reduced nephrotoxicity compared to monitoring trough concentrations, but there was no significant difference in clinical efficacy. One prospective multicenter observational study demonstrated no significant correlation between trough concentration and curative effect, but there was a correlation between trough concentration and nephrotoxicity in adults (32). However, the association between trough concentration or AUC_0–24_ and clinical results in children remains unclear (33). Hahn et al. (34) showed that the relationship between vancomycin AUC_0–24_/MIC and treatment failure or success could not be established in children with MRSA bacteremia due to limitations in the number of their study samples. Our study revealed that steady-state trough concentration, steady-state peak concentration and AUC_0–24_ shared no significant correlation with vancomycin treatment efficacy or bacterial clearance effect in children with severe infection at a median daily dose 40 mg/kg d, while higher steady-state trough concentrations and AUC_0–24_ were closely correlated with nephrotoxicity.

Children hospitalized in the PICU exhibit different pathophysiological characteristics than children not in the PICU. They exhibited more severe infection and reduced immune function and were more prone to organ failure. In addition, the combined use of multiple drugs increased the possibility of acute renal damage. AUCs measured during the first or second 24 h and lower than approximately 650 mg × hour/L may result in a decreased risk of AKI in adults (35). To date, few studies have focused on the AUC_0–24_ threshold in children. In 2015, a retrospective cohort study conducted by Le et al. (36) showed that vancomycin AUC_0–24_ ≥ 800 mg.h/L was independently associated with an increase in nephrotoxicity risk > 2.5-fold in children. Our study found that predicting nephrotoxicity using a binary logistic regression model of AUC_0–24_ may have higher efficiency than the trough concentration model. Therefore, regular TDM and monitoring of renal function during vancomycin administration are important for reducing renal impairment.

The 2020 IDSA guidelines (17) recommended setting a goal for children suspected or diagnosed with severe MRSA infection in which an AUC_0–24_/MIC ratio of 400–600 mg.h/L should be achieved, which was determined by analogy with previous studies conducted in adults. The AUC_0–24_ threshold in our study was far less than 800 mg.h/L and close to the threshold of 600 mg.h/L for adults. Our study demonstrated that AUC_0–24_ can be used for TDM to reduce the risk of nephrotoxicity. In addition, AUC_0–24_ < 537.18 mg.h/L may be a TDM strategy to be considered on the premise of ensuring the treatment effect in the clinic and avoiding nephrotoxicity.

Binary logistic regression analysis of nephrotoxicity showed that a longer treatment course of vancomycin, a higher trough concentration and a higher AUC_0–24_ were independent risk factors for nephrotoxicity. The daily dose in the nephrotoxicity group was lower than other groups in our study, which might be related to the corresponding adjustment according to the degrees of renal function damage and the clinical trough concentration. When renal function damage occurs clinically, a higher trough concentration or AUC_0–24_ range can be reached with a lower daily dose, so the dose of vancomycin needs to be flexibly adjusted. TDM (trough concentration or AUC_0–24_) should be performed to reduce the risk of nephrotoxicity when using vancomycin in the clinic. At the same time, physicians should closely monitor longer treatment courses and reduce the medication time to ensure the effect while reducing the risk of nephrotoxicity.

We compared the proportion of different trough concentrations corresponding to reaching the target AUC_0–24_. When the trough concentrations were between 10 and 20 mg/L, the AUC_0–24_ values of most children (39/40) were greater than 400 mg.h/L. When the trough concentrations were less than 10 mg/L, 48.3% (28/58) of children still reached the AUC_0–24_ level of 400–600 mg.h/L. Blindly increasing the drug dose to reach the trough concentration target value may increase the risk of nephrotoxicity. Consequently, the trough concentration needs to be monitored in combination with AUC_0–24_ to reduce the occurrence of nephrotoxicity.

One retrospective study showed that vancomycin trough concentration was not associated with treatment success or failure in pediatric patients with suspected Gram-positive infection, which was similar to our findings (37). The median APACHE II score of included pediatric patients was 17 in their study (37), which was lower than that of patients treated in PICU in our study. The patients was seriously ill, and the proportion of treatment failure and subsequent death in the hospital was 23.6% in present study. However, one systematic review and meta-analysis indicated that vancomycin trough concentrations of 10–15 mg/L was associated with significantly lower mortality in pediatric patients infected with Gram-positive pathogens (38). This finding seemed to be inspiring, while the high trough concentrations may incur serious adverse effects. Further research is needed to ensure the therapeutic effect of vancomycin and avoid adverse reactions in patients treated in the PICU.

There are several limitations to this study. First, this study was limited by factors that are inherent to the retrospective analysis and interpretation of data. Second, since our study is a single-center and retrospective study with small scales, the power of our results are restricted. Third, not every child had cultural evidence of Gram positive bacterial infection, so the AUC_0–24_/MIC value could not be calculated for each patient. In the future, multicenter prospective studies are needed to explore differences in the clinical efficacy and safety of vancomycin in the treatment of children with severe infection under the guidance of PK/PD parameters such as trough concentration and AUC_0–24_ and to further clarify the most appropriate AUC_0–24_ range of vancomycin for the treatment of children with severe infection. Finally, the mechanism of vancomycin metabolism differences in patients of different ages, weight and diseases is still not completely clear. Furthermore, the individual difference in the concentration of vancomycin and AUC at similar doses *in vivo* is also a problem perplexing clinicians. This study also can’t fully clarify the PK/PD of vancomycin in different children with severe infection in the PICU. The specific mechanism may need further studies.

## Conclusion

The steady-state trough concentration and AUC_0–24_ of vancomycin were not significantly correlated with the therapeutic efficacy or bacterial clearance effect when the daily dose of vancomycin was approximately 40 mg/kg d in critically ill children. However, the steady-state trough concentration and AUC_0–24_ were both closely correlated with nephrotoxicity. Therefore, the combination of AUC_0–24_ and trough concentration for TDM may reduce the risk of nephrotoxicity in pediatric populations.

## Data Availability Statement

The original contributions presented in this study are included in the article/supplementary material, further inquiries can be directed to the corresponding author/s.

## Ethics Statement

The studies involving human participants were reviewed and approved by the Medical Research Ethics Committee of Children’s Hospital Affiliated to Chongqing Medical University. Approval Document No. (2021) ethical review (study) No. (7). Written informed consent from the participants’ legal guardian/next of kin was not required to participate in this study in accordance with the national legislation and the institutional requirements.

## Author Contributions

CL and YF conceived the study, coordination and finalized the manuscript, and took responsibility for the article as a whole. BZ and WX participated in the design, data acquisition, database management, statistical analysis, and manuscript draft. KB, HD, JL, and FX participated in statistical analysis, database management, and manuscript draft. All authors contributed to the article and approved the submitted version.

## Conflict of Interest

The authors declare that the research was conducted in the absence of any commercial or financial relationships that could be construed as a potential conflict of interest.

## Publisher’s Note

All claims expressed in this article are solely those of the authors and do not necessarily represent those of their affiliated organizations, or those of the publisher, the editors and the reviewers. Any product that may be evaluated in this article, or claim that may be made by its manufacturer, is not guaranteed or endorsed by the publisher.
